# Role of bone morphogenetic proteins in form-deprivation myopia sclera

**Published:** 2011-03-08

**Authors:** Qing Wang, Guiqiu Zhao, Shichao Xing, Lina Zhang, Xian Yang

**Affiliations:** 1 Department of Ophthalmology, the Affiliated Hospital of Medical College, Qingdao University, Qingdao, China; 2 Department of Experimental Center of Molecular Biology, the Affiliated Hospital of Medical College, Qingdao University, Qingdao, China

## Abstract

**Purpose:**

To clarify the role of bone morphogenetic proteins (BMP-2,-4,-5) in sclera remodeling during myopia induction and their effect on sclera fibroblasts in cell culture.

**Methods:**

Reverse transcription and polymerase chain reaction (RT–PCR) as well as immunofluorescence were used to detect the expression of the BMPs in human and guinea pig posterior sclera. In guinea pig form-deprivation myopia (FDM) model, RT–PCR and western blotting were used to investigate changes of BMP expression in the posterior sclera. Human sclera fibroblast (HSF) was primarlly cultured and treated with various doses of BMP-2. Cell proliferation was evaluated by the MTT assay. RT–PCR and western-blot were used to determine the changes of collagen I, aggrecan, and possible activated signal pathway. Cell phenotype and activated signal pathway, especially for α-smooth muscle actin (α-SMA) and phospho-smad1/5/8 were then further investigated by cytoimmunofluorescence staining.

**Results:**

Both human and guinea pig sclera express BMP-2, −4, and −5. In FDM eyes, BMP-2 and BMP-5 expression were reduced in the posterior sclera. Cell proliferation increased significantly (p<0.05) and more cells differentiated into myofibroblast when incubated with 100 ng/ml BMP-2 . The expressions of collangen I, aggrecan, and phospho-smad1/5/8 significantly increased (p<0.05 respectively) as well.

**Conclusions:**

Various BMPs were expressed in human and guinea pig sclera. In the posterior sclera, the expressions of BMP-2 and BMP-5 decreased in FDM eyes. BMP-2 might be able to promote HSF proliferation and differentiation, as well as to help extracellular matrix synthesis potentially through classical Smad pathway.

## Introduction

Myopia is the most common visual disorder which affects approximately half of the world's young adult population [1-4]. The prevalence of myopia and the degree of severity is rapidly increasing, especially in some Asian communities [5]. Despite years of intensive research, the precise mechanisms which control ocular growth and development of refractive errors are still not well known. Postnatal scleral growth, like other connective tissues, is under the control of growth hormone or its downstream effectors, and there is a net loss of matrix in myopic sclera [6,7]. The role of transforming growth factor beta (TGF-β) in myopia was well investigated [8-12] during the past fifteen years. Bone morphogenetic protein (BMP), which belongs to transforming growth factor-beta superfamily, might play an important role in myopia development. BMPs were found in several ocular tissues and play an important role in eye development and differentiation [13]. Their other cellular functions, such as morphogenesis, cell proliferation, apoptosis, extracellular matrix synthesis etc., were reported [13] as well. However, to our best knowledge, the role of BMPs in homeostasis of the sclera has not been fully documented. The relationship between BMPs and axial elongation in myopia also remains obscure.

It is well known that sclera remodeling occurs during axial elongation in myopia [14,15] and scleral fibroblasts are the key cells in this process. The thinned posterior scleral in high myopia is associated with a general loss of type I collagen and aggrecan which accounting for most of scleral extra cellular matrix (ECM) [6,16]. Studies suggested that scleral cells comprised a subset of myofibroblasts [12,17]. One study showed that cultured with TGF-β brings about a rapid differentiation of sclera fibroblasts into α-smooth muscle actin (α-SMA)-expressing myofibroblasts [12].

In this study, we aim to clarify the role of BMPs (BMP-2, −4, and −5) by examining any changes during myopia induction in guinea pigs sclera, and in vitro the effect of BMPs on sclera fibroblasts differentiation, ECM, and its potential signal pathway.

## Methods

### Animals

All experiments undertaken conformed to the ARVO Statement for the Use of Animals in Ophthalmic and Vision Research and the United States NIH document “Guide for the Care and Use of Laboratory Animals 1996” were followed. Twenty pigmented guinea pigs (approximately 10~15 days old) were reared as previously reported [18]. Animals were housed in groups in a temperature controlled room (25 °C) in open topped cages, on a 12 h light (provided by ceiling fluorescent tubes 36W) and 12 h dark cycle, with free access to food (guinea pig pellets and fresh vegetables) and water.

### Experimental design and myopia induction

The animals were randomly assigned to 2 groups: diffusers (n=10) and normal controls (n=10). Diffuser-wear techniques employed in our experiments are similar to McFadden’s techniques [18]. The diffusers (white translucent hemispheric occluder with a diameter of 12 mm) were mounted on a matching plastic ring and glued to the periorbital fur of the eye for the whole two-week period of this experiment. The deprived eye in diffusers was randomly selected among animals and the fellow eye was untreated as a contralateral control.

### Biometric measurements

Biometric measurements include streak retinoscopy and ultrasonography. Retinoscopy was performed in a dark room by using a streak retinoscope and trial lenses when the eyes had been fully cyclopleged with 2 drops of tropicamide for approximately 15 min. The refraction was recorded as the mean value of the horizontal and vertical meridians. Axial length of the eye was measured under topical anesthesia by an A-scan ultrasonagraph with 11 MHz which had been used by Lu et al. [19]. Ocular refraction and axial length were collected at the beginning and end of the experiment.

### Tissue preparation

Human scleral tissues were excised from healthy adult human donor eyes (n=3; ages of 18, 19, and 25 years) obtained from the Eye Bank of the Affiliated Hospital of Medical College, Qingdao University, with the approval of Ethics Committee of Qingdao University (Qingdao, China) and complied with the tenets of the Declaration of Helsinki for biomedical research involving human subjects. Then each sclera was cut into equal halves along the optic disc and macula. The head of the optic nerve was discarded. One half was embedded with Optimum Cutting Temperature Compound (OTC; Sigma, St. Louis, MO), and cut into 8 μm sections at −20 °C. The other half was stored at −80 °C and used for RT–PCR. Sections were subsequently tiled onto slides (Corning Ltd, Tokyo, Japan), fixed with cool acetone for 15 min, air-dried, and kept frozen at −20 °C until use. Normal (n=10) and form deprived (n=10) guinea pigs were killed by overdoses of Chloral Hydrate. The eyeballs were removed and cut into halves about 1 mm posterior to the ora serrata on the ice plate. The anterior segment of the eye was discarded. The posterior sclera was excised by using a 6 mm-diameter trephine around the head of the optic nerve. The head of the optic nerve was discarded. The left sclera was flapped and stored at −80 °C.

### Human scleral cell isolation and culturing

Human scleral tissues were excised from surgical specimens collected during treatment for retinoblastoma (n=2, ages of 1.5 and 2 years) under signed informed consent obtained from donors and was also approved by the Ethics Committee of Qingdao University (Qingdao, China) and complied with the Tenets of the Declaration of Helsinki for biomedical research involving human subjects. The scleral tissue was cut into small pieces (about 1 mm^3^) and cultured in 25 mm^2^ flasks in Dulbecco’s modified Eagle’s medium (DMEM; Invitrogen, Carlsbad, CA)/Nutrient mixture F12 (1:1) with high glucose supplemented, 10% fetal bovine serum (FBS; Gibco) and incubated at 37 °C in a humidified incubator containing 5% CO_2_. When achieve a heavy primary monolayer, the cells were trypsinized for 3 min at room temperature in 0.25% trypsin/EDTA solution in phosphate buffered saline (PBS, Gibco) and subcultured at a split ratio of 1:3 in a 25 mm^2^ flask (Corning Ltd., Lowell, MA). The third passage of fibroblasts was used for this experiment.

### Cell proliferation assays

Cell proliferation was measured by 3-(4, 5-dimethylthiazol-2-yl) −2,5- diphenyltetrazolium bromide (MTT, Gibco) assay. Cells were plated in 96 well plates (Corning Ltd) with DMEM in 10% FBS at a 4×10^3^/cm^2^ density. Before treated with BMP-2, cells were synchronized by replacing the medium with serum-free medium for 24 h. Then cells were incubated with different concentrations (1 to 100 ng/ml) of BMP-2 at 37 °C for 1 to 6 successive days. This dose range of BMP-2 was commonly used in culture for in vitro studies although the normal physiologic levels are lower in bone at 1 to 2 ng/g. The medium was semi-replaced daily. On the seventh day, cells were washed twice with 10 mmol/l PBS (pH 7.2) and incubated with 0.5 mg/ml MTT at 10 μl/well for the last 4 h before the reaction was terminated with the addition of 150 μl dimethyl sulfoxide (DMSO, Sigma). The absorbance was determined at 490 nm using an enzyme linked immunosorbent assay (ELISA) reader (BIO-TEK Instruments, Winooski, VT).

### Total RNA isolation, reverse transcription and polymerase chain reaction (RT–PCR)

Human sclera cells were seeded in 6-well culture plates at 4×10^5^ cells/well and cultured for 24 h. After synchronized with serum-free DMEM for another 24 h, cells were incubated in DMEM containing BMP-2 (100 ng/ml). The culture without BMP-2 acted as controls. On day 2, the culture media was changed with the same concentration of BMP-2 in each well. After 72 h incubation, cells were harvested for RNA extraction. Sclera was grinded in liquid nitrogen. Total RNA were extracted from sclera (normal human sclera n=3;normal guinea pigs sclera n=3; form deprived guinea pig sclera n=5; contralateral control sclera n=5) and cells with TRIzol reagent (TaKaRa, Dalian, China) and contaminating DNA was digested using DNase I (Promega, Madison, WI) according to the manufacturer's protocol, and quantified using ultraviolet spectrophotometry by measuring OD260 and OD280 (optical density 260/280 higher than 1.9). cDNA was synthesized with 5 µg total RNA, 2 µl random 6 mers, 0.5 µl Oligo dT Primer, 2 µl PrimeScriptTM Buffer, and 0.5µl PrimeScript^TM^ RT Enzyme Mix I (TaKaRa) at 37 °C for 15 min. The reaction was suspended at 85 °C for 5 s. Conditions for PCR were 95 °C for 30 s, 35 cycles of 95 °C for 5 s, 55 °C (56 °C for BMP-3, BMPR-lB, and Smad1, respectively) for 30 s, and 72 °C for 30 s. The final extension step was at 72 °C for 7 min. The PCR products were then electrophoresed on a 2% agarose gel containing 10× Gelred reagents in parallel with 50 bp DNA markers. The nucleotide sequences of the primers used in the experiments are denoted in Table 1. β-actin (*Actb*) was used as an internal control. All band intensities were evaluated by densitometry (Gel-Pro Analyzer 4.5; Gel-Pro, Bethesda, MD). The ratio of a target gene versus *Actb* was computed as a series of numbers where there might be the smallest number. To make the expression trend clear, the value in the figures was the ratio of the series of numbers versus the smallest one. Because guinea pigs nucleotide sequences haven’t been reported, we designed probes to cover regions where the human and rat *BMP* sequences show high homology. It is therefore reasonable to expect that our primers specifically recognize the examined *BMP*s. To prove the identity, all the experiments were performed at least 3 times and the products were sequenced by TaKaRa.

**Table 1:** Sequences, annealing temperatures (Tm), and predicted product sizes of the primers used.

**Gene**	**Upstream primer**	**Downstream primer**	**Tm (°C)**	**Size (bp)**	**NCBI accession number (rat and human)**
BMP-2	GCGGAAACGCCTTAAGTCCA	GTGGAGTTCAGATGATCAGC	55	156	NM_017178.1 , NM_001200.2
BMP-3	TTGGCTGGAGCGAATGGATTA	GCTCAGGAATCCCAGAGACGAC	56	159	NM_017105.1 , NM_001201.2
BMP-4	TTTGTTCAAGATTGGCTCCCAAG	AAACGACCATCAGCATTCGGTTA	55	101	NM_012827.2 , BC020546.2
BMP-5	TCCACAGAACAATTTGGGCTTACA	ACCATGAACGGCTGCTTTGAC	55	120	NM_001108168.1 , NM_021073.2
BMP-6	CGCCTTCCTCAACGACCGCGG	GGAATCTGGGATAAGTTGAA	55	120	NM_013107.1 , NM_001718.4
BMP-7	TCCGGTTTGATCTTTCCAAGA	CCCGGATGTAGTCCTTATAGATCCT	55	81	NM_001191856.1 , NM_001719.2
BMPR-IA	GCCGTTTTGAAGCTGATGTCA	TCTTTGCGAGCGTCTTCTTGA	55	88	S75359.1 , NM_004329.2
BMPR-IB	TGGCTGACATGTACAGCTTTGG	GGCACTCGTCACTGCTCCAT	56	198	NM_001024259.1 , D89675.1
BMPR-ll	ACTGCAGATGGACGCATGG	AATCTCGATGGGAAATTGCAG	55	199	NM_080407.1 , NM_001204.6
Smad1	CTCCAATGTTAACCGGAACTCCAC	CTCTGCACGAAGATGCTGCTG	56	129	NM_013130.2 , U59423.1
Collagen I	TGCTGGCAAGAATGGCGATC	CTGTCTCAGCCTTGTCACCAC	55	122	NM_000088.3 , AF169346.1
aggrecan	GAAGTGATGCATGGCATTGAGG	ATGATGGCGCTGTTCTGAAGG	55	146	NM_022190.1 , BC036445.1
β-actin	GGCACCACACTTTCTACAATG	GGGGTGTTGAAGGTCTCAAAC	55	133	NM_031144.2 , NM_001101.3

### Western blot analysis

Human sclera cells were treated as described in RNA extraction. After incubated with 100 ng/ml BMP-2 for 72 h, cells were harvested for protein extraction. Form deprived guinea pig sclera (n=5) and contralateral control sclera (n=5) were grinded in liquid nitrogen. Cells and sclera tissues were homogenized separately in ice-cold extraction buffer (0.01 M Tris-HCl at pH 7.4, 0.15 M NaCl, 1% w/v Triton X-100, 0.1% SDS, 1% deoxycholic acid, 1mM EDTA) as well as protease inhibitors (1μM pepstatin, 1μg/ml leupeptin, and 0.2mM PMSF). After homogenization, samples were placed on ice for 30 min and centrifuged at 4 °C 12,000× g for 15 min. The supernatant was decanted and the precipitate was discarded. Protein concentrations were determined according to the BCA method using an Enhanced BCA Protein Assay Kit (Boster, Wuhan, China). Each protein sample (30 μg) was mixed with 5× sample buffer for SDS PAGE. The mixture was boiled for three min, electrophoresed on a 10% SDS polyacrylamide gel, and transferred to nitrocellulose membranes (Pall Corporation, East Hills, NY). Protein loading and transfer efficiency were monitored by staining the membranes with 1% Ponceau S. The membranes were washed three times with TBST (pH 7.6) and soaked in a blocking solution (5% w/v skim milk powder in 2.5 mM Tris-HCl and 14 mM NaCl plus 0.05% Tween-20) for 1 h at room temperature. The membranes were incubated overnight with primary antibodies at a 1:100 dilution (rabbit anti BMP-2, −4, and −5; 0.2ml; Boster, Wuhan, China) and 1:500 dilution (rabbit anti phospho-Smad1/5/8; 0.1 ml; , Millipore, Billerica, MA) at 4 °C in blocking solution. The membranes were then washed three times with TBST and incubated with a horseradish peroxidase-conjugated secondary antibody (goat anti-rabbit) at a 1:1,000 dilution (0.4 μg/ml; Boster) for another 1 h at room temperature. The membranes were again washed three times with TBST. The reaction products were visualized with BeyoECL Plus western blotting detection reagents (Beyotime Institute of Biotechnology, Haimen, China). Images were captured with a Fuji Film LAS3000 imaging system and analyzed with MultiGauge software (Fuji Film, Tokyo, Japan). β-actin (Kang Chen, China) was used as a housekeeping protein to normalize the protein load.

### Immunofluorescent staining

The slides were washed three times with PBS, nonspecific bindings were blocked by incubation in 10% (v/v) goat serum in PBS for 30 min at room temperature. Then the slides were incubated at 4 °C overnight with primary antibodies against BMP-2, −4, and-5 (BA0585; BA0662; BA0663; 1:200 dilution; Boster) diluted in PBS. The antibody-treated and negative control slides were washed with PBS three times at room temperature, then incubated with FITC labeled goat anti-rabbit IgG antibodies (BA1105; Boster) diluted in PBS at room temperature for 1 h. Fluorescent signals were detected using a fluorescent microscope (TS-100; Nikon, Tokyo, Japan).

Cells grown on 96-well plates were fixed with 4% (v/v) paraformaldehyde for 30 min and then made permeable with 0.1% Triton X-100 in PBS for 10 min. Nonspecific binding was blocked by incubation in 10% (v/v) goat serum in PBS for 30 min at room temperature. Following incubation of the cells with primary antibody against phospho-Smad1/5/8 (AB3848; Millipore), α-SMA (BM0002; Boster) diluted in PBS at 4 °C overnight. Cells were then probed with FITC-labeled (BA1105; Boster) or Cy3-labeled goat anti-rabbit (BA1032; Boster) secondary antibodies diluted in PBS and incubated at the room temperature for another 1 h. Nuclear staining with DAPI was applied. Fluorescent signals were detected using a fluorescent microscope (TS-100; Nikon).

### Statistical analysis

Data were expressed as mean±standard deviation (SD). Differences between the groups were compared by using a one-way ANOVA (ANOVA) with a Tukey post hoc test or Bonferroni Correction. The Student’s *t*-test was performed for statistical analysis of cytometry between the experiment and control groups. Statistical analysis was performed using the SPSS 15.0 statistical software (IBM, Chicago, IL). The results were considered to be statistically significant at p<0.05.

## Results

### Expression of BMPs and BMP receptors in human and guinea pig sclera

**Figure 1:**
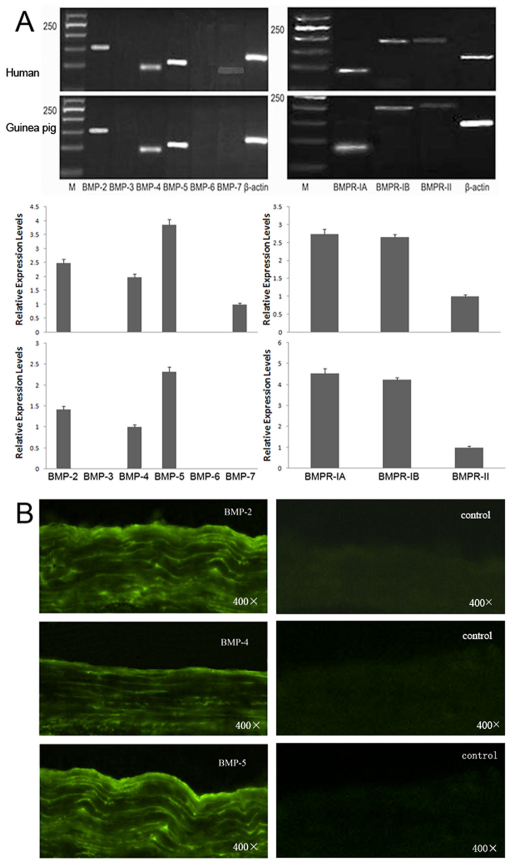
Expressions of BMPs and BMP receptors in human and guinea pig sclera. **A**: Semi-quantitative RT–PCR analysis of total RNA from 3 normal human sclera and 3 normal guinea pig sclera using specific primers for *BMP-2* to *BMP-7*, *BMPR-IA*, *BMPR-IB*, and *BMPR-II* normalized to *Actb* at 35 cycles (M: molecular size marker). **B**: Distribution of the BMPs in human sclera by indirect immunofluorescence (FITC marked the secondary antibody).

In normal human and guinea pig sclera, BMP-2, −4, −5, and all the BMPRs could be detected after RT–PCR (Figure 1A). The relative expression level of *BMP-5* was strongest, followed by *BMP-2* and *BMP-4*. The expression of *BMPR-IA* and *BMPR-IB* were higher than that of *BMPR-II*. The expression of BMPs in human sclera were further confirmed by immunofluorescence (Figure 1B).

### The expression of BMPs in guinea pig sclera of form-deprivation myopia model

**Figure 2:**
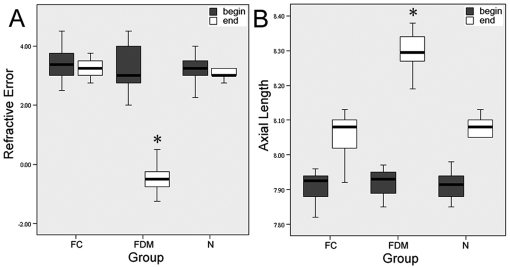
Differences in refractive error and axial length after induction of myopia. Differences in refractive error (**A**) and axial length (**B**) among form-deprived myopic (FDM), contralateral control (FC) and normal control(N) eyes of guinea pigs were illustrated after two weeks. The asterisk denotes a p<0.001 versus FC and N eyes.

The differences between the two eyes of each animals in axial length were not significant for the two groups at the beginning (p=0.794, one-way ANOVA with Bonferroni correction, Figure 2). At the 14th day, monocularly deprived eyes had myopia of −0.48±0.51 D and an axial length of 8.29±0.05 mm which was significantly more myopic and longer than either the contralateral control eyes (+3.22±0.34 D; t=-12.814,df=18, p<0.001; 8.05±0.06 mm, t=7.23,df=18 p<0.001) or age-matched normal eyes (+3.07±0.54 D, t=-11.878, df=18, p<0.001; 8.06±0.06 mm, t=9.084, df=18, p<0.001, independence samples test, Figure 2).

**Figure 3:**
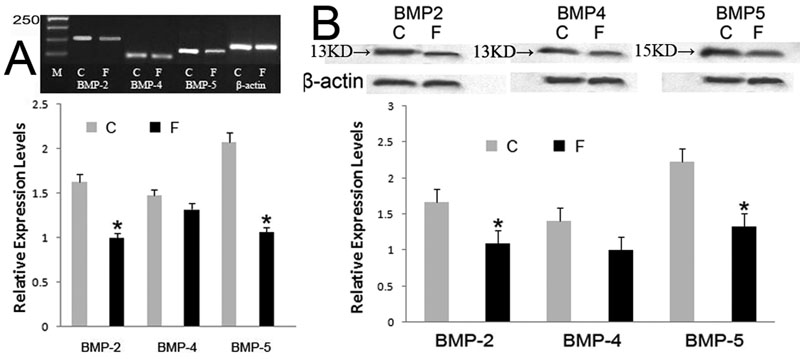
Effect of myopia induction on the expressions of BMPs in guinea pigs sclera. **A**: Significant decreases in mRNA expression were found in *BMP-2* and *BMP-5* compared with the internal control eyes. C: contralateral control, F: form-deprivation myopia. **B**: western blotting also showed a significant decrease for BMP-2 and BMP-5 but not for BMP-4. Bar graph shows changes in mRNA and protein expressions of BMP subtypes in the posterior sclera during form-deprived myopia in guinea pigs. Values (mean±standard error of the mean) were expressed as relative expression levels. The asterisk denotes a p<0.05.

The relative expression levers of FDM eyes were significantly lower than contralateral control eyes for BMP-2 (p=0.017) and BMP-5 (p=0.028). However, the expression of BMP-4 was not significantly altered in FDM eyes (p=0.162; Figure 3A). The identity of PCR products was confirmed by sequence analysis of vector-cloned cDNA fragments. Negative control experiments without template did not yield any products. The protein expressions of BMP-2, −4 and −5 could be detected by western blot (bands at about 13 kDa and 15 kDa, Figure 3B). After 14 days of visual deprivation, significant decrease was shown for BMP-2 (−32.65%, p=0.026) and BMP-5 (−25.11%, p=0.034) compared with the contralateral control eyes of the same animals, while no significant changes shown for BMP-4 (p=0.158; Figure 3B).

### The effect of BMP-2 on HSF in vitro

**Figure 4:**
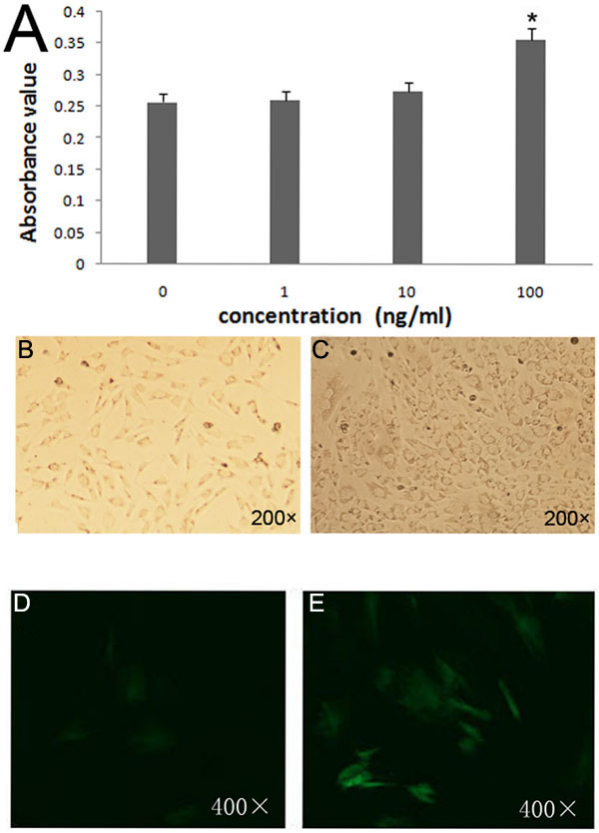
The effect of BMP-2 on HSF in vitro. **A**: MTT Assay of the effect of BMP-2 on HSF proliferation at different concentration on 6th day. Absorbance value at a wavelength of 490 nm. HSF proliferation in the presence of BMP-2 (100 ng/ml) was significantly higher on day 6 than BMP-2 with other concentrations or controls (p<0.05, n=6). The asterisk *stands for p<0.05. The morphology of primary cultured human scleral cells (**B**) the cells not exposed with BMP-2; (**C**) the cells cultured with 100 ng/ml BMP-2 on day 6. Cultured scleral fibroblasts were incubated without (**D**) or with (**E**) BMP-2(100ng/ml) for 6 days. The expression of the myofibroblast-marker, α-SMA was assessed using fluorescent immunocytochemistry (FITC-labeled the second anitibody).

The scleral cells began growing out from pieces of sclera tissue after almost 10 days in culture. They exhibited a fibroblast-like spindle shape or polygonal shape in morphology for scleral cells, which grew in a vortex pattern cultured in monolayer. After exposed with various concentrations of BMP-2 (0, 1, 10, and 100 ng/ml) for one to seven days, cells proliferation was significantly higher on day 6 with 100 ng/ml BMP-2 (Figure 4A) than other concentrations, and the shape of cells was polygonal (Figure 4B,C). And there was a rapid differentiation of fibroblasts into α-SMA-expressing myofibroblasts with the addition of 100 ng/ml BMP-2 in cell cultures (Figure 4D,E).

**Figure 5:**
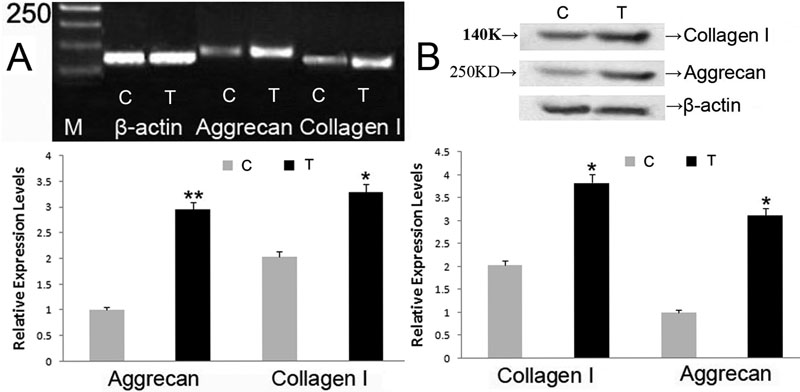
Effect of BMP-2 on production of collagen I and aggrecan. The mRNA (**A**) and protein (**B**) expression levels for collagen I and aggrecan were significantly increased with 100 ng/ml BMP-2, 72 h in vitro. Bar graphs revealed changes in mRNA and protein expressions (mean±standard error of the mean) where values were stated as relative expression levels. C: control group, T: BMP-2 treatment group. The asterisk stands for p<0.05 and the double asterisk stands for p<0.01.

As shown in Figure 5A,B, the expressions of aggrecan and collagen l as well as their protein expression levels were significantly increased when being cultured with 100 ng/ml BMP-2 for 72 h. The time point was selected according to Hu et al. [20] and Yoon et al. [21].

### Potential signal pathway of BMP effects in vitro

**Figure 6:**
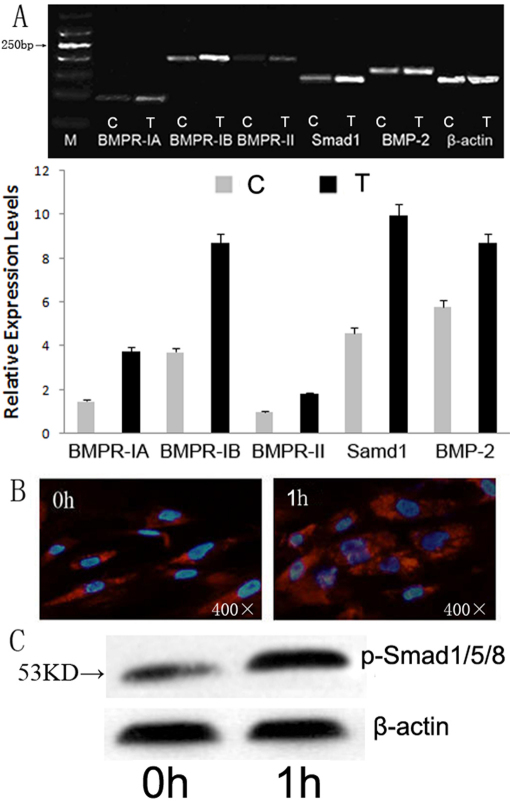
Potential effect of BMP-2 on the signal pathway in vitro. **A**: The mRNA expression in *BMPR*s, *smad1*, and *BMP-2* after 24 h of stimulation with 100 ng/ml BMP-2. **B**: Immunofluorescent staining of Smad1/5/8 phosphorylation located in the cells treated by 100 ng/ml BMP-2 for 1 h.The second antibody is Cy3-labeled goat anti-rabbit (Boster) and nuclear was stained with DAPI. **C**: The protein level of phospho-Smad1/5/8 in the treated cells detected by western blot.

The expression of BMPR-IA, -IB, -II, Samd1, and BMP-2 significantly increased in vitro (Figure 6A) after cells were exposed with100 ng/ml BMP-2 for 24 h. The expression level of BMPR-IA and BMPR-IB were higher than BMPR-II.

To confirm the effect of BMP-2 on signal pathway in vitro, cells were stained with immunofluorescence labeled Smad1/5/8 and detected by western blotting also. After incubated with100 ng/ml BMP-2 for 1 h, it showed more marked fluorescent signals localized in the nucleus of the treated cells than untreated cells (Figure 6B). Western blots also showed a higher phospho-Smad1/5/8 protein level in the treated cells (Figure 6C).

## Discussion

In this study, the expression of selected BMPs were investigated in human and guinea pig sclera as well as in form-deprivation myopia sclera of guinea pigs. To our best knowledge, this is the first report about the expression and functional role of BMPs in guinea pigs sclera, and particularly for a form-deprivation myopia model.

The present study showed that both human and guinea pig sclera expressed BMP-2, −4, and −5. The additional expression of BMP receptors (BMPR-IA, -IB, and -II) in sclera indicates that BMPs play a role in normal sclera homeostasis. In the following study, there is a significant downward regulation of BMP-2 and −5 in the scleral remodeling during myopia induction, especially BMP-2. BMP-2 is expressed in the human cornea and is considered to be a heparin-binding cytokine that can modulate corneal fibroblast apoptosis [22]. It was also found to promote sclera fibroblast proliferation in vitro [20]. McGlinn et al. [23] reported that chicks following 6 h and 3 days of diffuser-wear, *BMP-2* mRNA levels were significantly down-regulated in the retina and RPE. These research works show that there could be an additional role for BMP-2 in eye tissue. In a study [24]of possible mechanisms of scleral remodeling in the development of myopia found that there were mechanical stresses induced *BMP-2* mRNA expression in human scleral fibroblasts after 30 min and 24 h. Another study [25] also reported that variations in the *BMP2K* gene are strongly correlated with high myopia in the Taiwanese population. In our study, BMP-2 was decreased in posterior sclera of myopia eyes, which would further indicate the BMP-2 is involved in the growth of human sclera.

It is reported that BMP-5 is also expressed in adult cornea [26]. BMP-5 expression increases during chondrocyte differentiation both in vivo and in vitro and this could promote proliferation and cartilage matrix synthesis through smad1/5/8 and p38 MAP kinase pathway [27]. In synovial tissue of patients with osteoarthritis and rheumatoid arthritis, the expression of BMP-5 was decreased [28]. In our study, there is a very high expression of BMP-5 in normal guinea pigs sclera and a significantly decreased expression in FDM. The decreased expression of BMP-5 indicates that BMP-5 was involved in sclera remodeling during myopia induction.

Other studies found BMP-4 could block the induction of fibronectin by TGF-β2 in TM cells [29]. In primary open angle glaucoma, elevated BMP antagonist expressed by TM cells inhibits BMP-4 antagonism of TGF-β2 and this inhibition leads to increased ECM deposition and elevated IOP [13]. In humans, mutations in BMP-4 [30] and BMP-7 [31] have been reported to be associated with eye and brain developmental anomalies, including the development of myopia in specific families. However, in our study, it did not show significant changes in BMP-4 expression during myopia induction. Further research should be focused on the relationship of BMP-4 and its antagonist with the effect of TGF-β in sclera ECM remodeling.

It was reported that HSF proliferation and scleral extracellular matrix remodeling events result in changes in biomechanical properties of the sclera and subsequent changes in axial length [24,32,33]. FDM significantly decreased total cell numbers in the region between the optic nerve and 10 degrees nasal (equivalent to myopic crescent location in humans) compared with control or normal eyes [34]. In our study, we used BMP-2 to clarify the effect of BMPs on HSF differentiation, extracellular matrix synthesis and its potential signal pathway in human sclera cells in vitro. It showed that BMP-2 significantly promoted HSF cell proliferation. Cell morphology and organization were also changed with the increasing concentration of BMP-2. The highest concentration of BMP-2 we had conducted is 100 ng/ml, for it is the most concentration used in vitro. For cell aggregation and polygonal could related to rapidly increasing cell differentiation and expression of more adhesion molecules. The increasing expression of α-SMA suggested that several HSF had differentiated into myofibroblasts. Myofibroblasts are generally defined as highly contractile cells that express smooth muscle protein, α-SMA [35] and comprise as a subset of scleral cells. One study suggested an age-dependent increase in the proportion of myofibroblasts [14,36]. Exposure of sclera cells to the reduced levels of TGF-β which was found in form-deprivation myopia sclera will decrease cell-mediated contraction and reduce α-SMA expression [12]. Myofibroblasts are capable of modifying their extracellular environment both through contraction and production of new extracellular matrix. Our result of increased myofibroblasts indicates that there are increased cell-mediated contractions with BMP-2.

Type I collagens constitute the majority of the collagens (approximately 99%) in human sclera and aggrecan is a major component of sclera proteoglycans [6]. In myopia, the posterior sclera remodeling was characterized by physical loss of the scleral extracellular matrix which were mainly due to the reduced production and increased degradation of type I collagen and aggrecan [6,37]. Previous studies had shown an increased expression of tissue inhibitor of matrix metalloproteinase (TIMP-2) and a decreased expression of MMP-2 in HSF after incubation with BMP-2 [20]. It implies that BMP-2 could decrease the degradation of the extracellular matrix of sclera. In this study, we observed significantly increased expression of aggrecan and collagen when cells incubated with 100 ng/ml BMP-2. This is different from previous studies which BMP-2 could not change mRNA of Type I collagen at any doses in osteoinductive cells such as intervertebral disc cells or chondrocyte [38,39]. Our results further suggested that there was an increased extracellular matrix production with 100 ng/ml BMP-2, which were similar to the results of Seko et al. [40].

BMPs exert their biologic effects through binding to the cell-surface serine-threonine kinase receptors BMP-RI and BMP-RII. This activation leads to phosphorylation of intracellular signaling molecules including Smad1, Smad5, and Smad8 [41,42]. Smad1/5/8 are receptor-activated Smads (R-Smads) which were phosphorylated by BMP type I receptors. After phosphorylation, R-Smads are released rapidly from the Type-I receptor to interact with Co-Smads (Smad4) and form hetero-oligomeric complexes which then translocate into the nucleus to regulate the transcription of various target genes [43]. In our study, BMPRs were known to be functional because Smad1/5/8 phosphorylation was observed following addition of BMP-2 (100 ng/ml).

A increased expression of mRNA for Smad1 and protein levels of phospho-Smad1/5/8 indicate a classic Smad signal pathway might be activated in HSF with BMP-2 stimulation. To further prove it, more experiments should be done as other proteins may be involved in the effects of activated BMP receptors on cell proliferation or matrix synthesis.

During myopia development, the biomechanical properties of the sclera are altered. Although scleral matrix remodelling was considered to be the sole determinant of this kind of change, but the importance of scleral cells, particularly scleral myofibroblasts should not be overlooked [7]. Our data suggest that BMP-2 could influence fibroblast proliferation and differentiation as well as extracellular matrix synthesis, which contributes to the development of human myopia. Future research is needed to investigate how BMP-2 controls ocular growth in animal models and influences on the remodeling of the sclera.

In conclusion, BMP-2, -4, and -5 are expressed in human and normal guinea pigs sclera. For myopia induction, the changes of their expression were found in posterior sclera of guinea pig. It suggests that BMPs might play an important role in sclera homeostasis and be potential candidates for myopia control.

## References

[1] Fan DS, Lam DS, Lam RF, Lau JT, Chong KS, Cheung EY, Lai RY, Chew SJ (2004). Prevalence, incidence, and progression of myopia of school children in Hong Kong.. Invest Ophthalmol Vis Sci.

[2] Jobke S, Kasten E, Vorwerk C (2008). The prevalence rates of refractive errors among children, adolescents, and adults in Germany.. Clin Ophthalmol.

[3] Rose K, Smith W, Morgan I, Mitchell P (2001). The increasing prevalence of myopia: implications for Australia.. Clin Experiment Ophthalmol.

[4] Liang YB, Wong TY, Sun LP, Tao QS, Wang JJ, Yang XH, Xiong Y, Wang NL, Friedman DS (2009). Refractive errors in a rural Chinese adult population the Handan eye study.. Ophthalmology.

[5] Seet B, Wong TY, Tan DT, Saw SM, Balakrishnan V, Lee LK, Lim AS (2001). Myopia in Singapore: taking a public health approach.. Br J Ophthalmol.

[6] Rada JA, Shelton S, Norton TT (2006). The sclera and myopia.. Exp Eye Res.

[7] McBrien NA, Cornell LM, Gentle A (2001). Structural and ultrastructural changes to the sclera in a mammalian model of high myopia.. Invest Ophthalmol Vis Sci.

[8] Honda S, Fujii S, Sekiya Y, Yamamoto M (1996). Retinal control on the axial length mediated by transforming growth factor-beta in chick eye.. Invest Ophthalmol Vis Sci.

[9] Seko Y, Shimokawa H, Tokoro T (1995). Expression of bFGF and TGF-beta 2 in experimental myopia in chicks.. Invest Ophthalmol Vis Sci.

[10] Jobling AI, Nguyen M, Gentle A, McBrien NA (2004). Isoform-specific changes in scleral transforming growth factor-beta expression and the regulation of collagen synthesis during myopia progression.. J Biol Chem.

[11] Jobling AI, Wan R, Gentle A, Bui BV, McBrien NA (2009). Retinal and choroidal TGF-beta in the tree shrew model of myopia: isoform expression, activation and effects on function.. Exp Eye Res.

[12] Jobling AI, Gentle A, Metlapally R, McGowan BJ, McBrien NA (2009). Regulation of scleral cell contraction by transforming growth factor-beta and stress: competing roles in myopic eye growth.. J Biol Chem.

[13] Wordinger RJ, Clark AF (2007). Bone morphogenetic proteins and their receptors in the eye.. Exp Biol Med (Maywood).

[14] McBrien NA, Lawlor P, Gentle A (2000). Scleral remodeling during the development of and recovery from axial myopia in the tree shrew.. Invest Ophthalmol Vis Sci.

[15] McBrien NA, Gentle A (2003). Role of the sclera in the development and pathological complications of myopia.. Prog Retin Eye Res.

[16] McBrien NA, Jobling AI, Gentle A (2009). Biomechanics of the sclera in myopia: extracellular and cellular factors.. Optom Vis Sci.

[17] Poukens V, Glasgow BJ, Demer JL (1998). Nonvascular contractile cells in sclera and choroid of humans and monkeys.. Invest Ophthalmol Vis Sci.

[18] McFadden SA, Howlett MH, Mertz JR (2004). Retinoic acid signals the direction of ocular elongation in the guinea pig eye.. Vision Res.

[19] Lu F, Zhou X, Jiang L, Fu Y, Lai X, Xie R, Qu J (2009). Axial myopia induced by hyperopic defocus in guinea pigs: A detailed assessment on susceptibility and recovery.. Exp Eye Res.

[20] Hu J, Cui D, Yang X, Wang S, Hu S, Li C, Zeng J (2008). Bone morphogenetic protein-2: a potential regulator in scleral remodeling.. Mol Vis.

[21] Yoon ST, Kim KS, Li J, Park JS, Akamaru T, Elmer WA, Hutton WC (2003). The effect of bone morphogenetic protein-2 on rat intervertebral disc cells in vitro.. Spine (Phila Pa 1976).

[22] Kim WJ, Mohan RR, Mohan RR, Wilson SE (1999). Effect of PDGF, IL-1alpha, and BMP-2/4 on corneal fibroblast chemotaxis: expression of the platelet-derived growth factor system in the cornea.. Invest Ophthalmol Vis Sci.

[23] McGlinn AM, Baldwin DA, Tobias JW, Budak MT, Khurana TS, Stone RA (2007). Form-deprivation myopia in chick induces limited changes in retinal gene expression.. Invest Ophthalmol Vis Sci.

[24] Cui W, Bryant MR, Sweet PM, McDonnell PJ (2004). Changes in gene expression in response to mechanical strain in human scleral fibroblasts.. Exp Eye Res.

[25] Liu HP, Lin YJ, Lin WY, Wan L, Sheu JJ, Lin HJ, Tsai Y, Tsai CH, Tsai FJ (2009). A novel genetic variant of BMP2K contributes to high myopia.. J Clin Lab Anal.

[26] You L, Kruse FE, Pohl J, Völcker HE (1999). Bone morphogenetic proteins and growth and differentiation factors in the human cornea.. Invest Ophthalmol Vis Sci.

[27] Mailhot G, Yang M, Mason-Savas A, Mackay CA, Leav I, Odgren PR (2008). BMP-5 expression increases during chondrocyte differentiation in vivo and in vitro and promotes proliferation and cartilage matrix synthesis in primary chondrocyte cultures.. J Cell Physiol.

[28] Bramlage CP, Häupl T, Kaps C, Ungethüm U, Krenn V, Pruss A, Müller GA, Strutz F, Burmester GR (2006). Decrease in expression of bone morphogenetic proteins 4 and 5 insynovial tissue of patients with osteoarthritis and rheumatoid arthritis.. Arthritis Res Ther.

[29] Wordinger RJ, Fleenor DL, Hellberg PE, Pang IH, Tovar TO, Zode GS, Fuller JA, Clark AF (2007). Effects of TGF-beta2, BMP-4, and gremlin in the trabecular meshwork:implications for glaucoma.. Invest Ophthalmol Vis Sci.

[30] Bakrania P, Efthymiou M, Klein JC, Salt A, Bunyan DJ, Wyatt A, Ponting CP, Martin A, Williams S, Lindley V, Gilmore J, Restori M, Robson AG, Neveu MM, Holder GE, Collin JR, Robinson DO, Farndon P, Johansen-Berg H, Gerrelli D, Ragge NK (2008). Mutations in BMP4 cause eye, brain, and digit developmental anomalies:overlap between the BMP4 and hedgehog signaling pathways.. Am J Hum Genet.

[31] Wyatt AW, Osborne RJ, Stewart H, Ragge NK (2010). Bone morphogenetic protein 7 (BMP7) mutations are associated with variable ocular, brain, ear, palate, and skeletal anomalies.. Hum Mutat.

[32] Rada JA, Johnson JM, Achen VR, Rada KG (2002). Inhibition of scleral proteoglycan synthesis blocks deprivation-induced axial elongation in chicks.. Exp Eye Res.

[33] Rada JA, Achen VR, Penugonda S, Schmidt RW, Mount BA (2000). Proteoglycan composition in the human sclera during growth and aging.. Invest Ophthalmol Vis Sci.

[34] Backhouse S, Phillips JR (2010). Effect of induced myopia on scleral myofibroblasts and in vivo ocular biomechanical compliance in the Guinea pig.. Invest Ophthalmol Vis Sci.

[35] Hinz B, Celetta G, Tomasek JJ, Gabbiani G, Chaponnier C (2001). Alphasmooth muscle actin expression upregulates fibroblast contractile activity.. Mol Biol Cell.

[36] Poukens V, Glasgow BJ, Demer JL (1998). Nonvascular contractile cells in sclera and choroid of humans and monkeys.. Invest Ophthalmol Vis Sci.

[37] McBrien NA, Gentle A (2003). Role of the sclera in the development and pathological complications of myopia.. Prog Retin Eye Res.

[38] Li J, Yoon ST, Hutton WC (2004). Effect of bone morphogenetic protein-2 (BMP-2) on matrix production, other BMPs, and BMP receptors in rat intervertebral disc cells.. J Spinal Disord Tech.

[39] Kuh SU, Zhu Y, Li J, Tsai KJ, Fei Q, Hutton WC, Yoon ST (2008). Can TGF-beta1 and BMP-2 act in synergy to transform bone marrow stem cells to discogenic-type cells?. Acta Neurochir (Wien).

[40] Seko Y, Azuma N, Takahashi Y, Makino H, Morito T, Muneta T, Matsumoto K, Saito H, Sekiya I, Umezawa A (2008). Human sclera maintains common characteristics with cartilage throughout evolution.. PLoS ONE.

[41] ten Dijke P, Korchynskyi O, Valdimarsdottir G, Goumans MJ (2003). Controlling cell fate by bone morphogenetic protein receptors.. Mol Cell Endocrinol.

[42] de Caestecker M (2004). The transforming growth factor-β superfamily of receptors.. Cytokine Growth Factor Rev.

[43] Nohe A, Keating E, Knaus P, Petersen NO (2004). Signal transduction of bone morphogenetic protein receptors.. Cell Signal.

